# The haplotype-resolved T2T genome assembly reveals the evolution and adaptation mechanisms of cold resistance in *Rosa beggeriana*

**DOI:** 10.1093/hr/uhag210

**Published:** 2026-06-03

**Authors:** Huayang Li, Xiang Wang, Yaping Kou, Longzhen Li, Kang Zhang, Xiaofei Wang, Ruidong Jia, Xin Zhao, Hong Ge, Feng Cheng, Shuhua Yang

**Affiliations:** State Key Laboratory of Vegetable Biobreeding, Institute of Vegetables and Flowers, Chinese Academy of Agricultural Sciences, Beijing 100081, China; Key Laboratory of Biology and Genetic Improvement of Flower Crops (North China), Ministry of Agriculture and Rural Affairs, Beijing 100081, China; State Key Laboratory of Vegetable Biobreeding, Institute of Vegetables and Flowers, Chinese Academy of Agricultural Sciences, Beijing 100081, China; Key Laboratory of Biology and Genetic Improvement of Horticultural Crops of the Ministry of Agriculture and Rural Affairs, Sino-Dutch Joint Laboratory of Horticultural Genomics, Beijing 100081, China; State Key Laboratory of Vegetable Biobreeding, Institute of Vegetables and Flowers, Chinese Academy of Agricultural Sciences, Beijing 100081, China; Key Laboratory of Biology and Genetic Improvement of Flower Crops (North China), Ministry of Agriculture and Rural Affairs, Beijing 100081, China; State Key Laboratory of Vegetable Biobreeding, Institute of Vegetables and Flowers, Chinese Academy of Agricultural Sciences, Beijing 100081, China; Key Laboratory of Biology and Genetic Improvement of Flower Crops (North China), Ministry of Agriculture and Rural Affairs, Beijing 100081, China; State Key Laboratory of Vegetable Biobreeding, Institute of Vegetables and Flowers, Chinese Academy of Agricultural Sciences, Beijing 100081, China; Key Laboratory of Biology and Genetic Improvement of Horticultural Crops of the Ministry of Agriculture and Rural Affairs, Sino-Dutch Joint Laboratory of Horticultural Genomics, Beijing 100081, China; State Key Laboratory of Vegetable Biobreeding, Institute of Vegetables and Flowers, Chinese Academy of Agricultural Sciences, Beijing 100081, China; Key Laboratory of Biology and Genetic Improvement of Flower Crops (North China), Ministry of Agriculture and Rural Affairs, Beijing 100081, China; State Key Laboratory of Vegetable Biobreeding, Institute of Vegetables and Flowers, Chinese Academy of Agricultural Sciences, Beijing 100081, China; Key Laboratory of Biology and Genetic Improvement of Flower Crops (North China), Ministry of Agriculture and Rural Affairs, Beijing 100081, China; State Key Laboratory of Vegetable Biobreeding, Institute of Vegetables and Flowers, Chinese Academy of Agricultural Sciences, Beijing 100081, China; Key Laboratory of Biology and Genetic Improvement of Flower Crops (North China), Ministry of Agriculture and Rural Affairs, Beijing 100081, China; State Key Laboratory of Vegetable Biobreeding, Institute of Vegetables and Flowers, Chinese Academy of Agricultural Sciences, Beijing 100081, China; Key Laboratory of Biology and Genetic Improvement of Flower Crops (North China), Ministry of Agriculture and Rural Affairs, Beijing 100081, China; State Key Laboratory of Vegetable Biobreeding, Institute of Vegetables and Flowers, Chinese Academy of Agricultural Sciences, Beijing 100081, China; Key Laboratory of Biology and Genetic Improvement of Horticultural Crops of the Ministry of Agriculture and Rural Affairs, Sino-Dutch Joint Laboratory of Horticultural Genomics, Beijing 100081, China; State Key Laboratory of Vegetable Biobreeding, Institute of Vegetables and Flowers, Chinese Academy of Agricultural Sciences, Beijing 100081, China; Key Laboratory of Biology and Genetic Improvement of Flower Crops (North China), Ministry of Agriculture and Rural Affairs, Beijing 100081, China

Dear Editor,

*Rosa beggeriana* is a Central Asiatic native ornamental shrub with extreme freezing tolerance, which has been applied to cold-tolerant rose breeding through interspecific hybridization. Over recent years, several rose genome assemblies have been released, revealing the mechanisms of genomics-based modern rose breeding and ornamental traits [1–3]. Despite the increasing availability of rose genomes, the practical utility of cold tolerance-associated genes unique to wild germplasm remains largely elusive. Our study addresses this gap by presenting complete and haplotype-resolved assemblies for cold resistance mechanisms and adaptation to habitats. This breakthrough elucidates the molecular mechanisms of cold tolerance in *R. beggeriana* and establishes a theoretical foundation for breeding cold-tolerant roses.

To obtain the high-quality genome of *R. beggeriana*, we generated 27.92 Gb (66.3× coverage) of HiFi PacBio Sequel II reads, 33.11 Gb (78.7× coverage) of Illumina data from the NovaSeq 6000 platform, and 67.90 Gb (165.5× coverage) of Hi-C data digested with HindIII restriction endonuclease. K-mer analysis estimated the genome size of *R. beggeriana* to be 420.86 Mb, with a heterozygosity rate of 1.05%. Owing to the observed relatively high heterozygosity in *R. beggeriana*, we employed the hifiasm (0.19.9-r616) for genome assembly and haplotype phasing, and utilized the juicer-1.6 and 3d-dna for anchoring seven chromosomes, yielding two distinct haplotypes consist of RbHap1 (417.13 Mb) and RbHap2 (421.87 Mb) (Fig. 1A). All seven chromosomes are gap-free, with seven centromeres and 14 telomeres determined (Fig. 1A). Quality assessment indicated this genome had high integrity and accuracy, with Benchmarking Universal Single-Copy Orthologs (BUSCO) completeness of 99.3% (Hap1) and 99.2% (Hap2), LTR assembly index (LAI) of 18.88 (Hap1) and 19.15 (Hap2), and consensus quality value of 66.92 (Hap1) and 66.52 (Hap2). The identified repetitive elements were 239.61 Mb (Hap1, 54.81%) and 239.89 Mb (Hap2, 55.57%) with masking across both haplotype assemblies. A total of 38,039 and 37,962 protein-coding genes were predicted in Hap1 and Hap2, respectively. Functional annotation was achieved for 92.48% (Hap1) and 92.51% (Hap2) of the predicted genes using the NR, Pfam, UniProt, GO, and KEGG databases. Various non-coding RNAs were detected, including 725 tRNAs, 1,047 rRNAs, 188 miRNAs, and 426 snRNAs in Hap1, and 756 tRNAs, 1,059 rRNAs, 165 miRNAs, and 379 snRNAs in Hap2. With synteny alignment and coordinate location approaches between the two haplotypes, 32,058 and 32,105 genes with homology and similar sites were defined as allelic genes. Among these, 15,267 and 15,248 genes, respectively, had allelic partners with identical CDS in the corresponding haplotypes (Fig. 1B). Additionally, 5,981 and 5,757 genes were identified in each haplotype that lacked an allelic counterpart, referred to as haplotype-specific genes. In the low-temperature treatment transcriptome, most cold-responsive genes belonged to allelic and bi-allelic genes categories, whereas only 471 and 465 haplotype-specific genes were functional in Hap1 and Hap2, respectively. The expression levels of haplotype-specific genes were lower than those of allelic genes, while the levels of bi-allelic genes were higher than those of allelic genes in various tissues. Functional enrichment analysis highlighted the role of haplotype-specific genes, which are significant in biological pathways related to stress response and catalytic activity.

**Figure 1:**
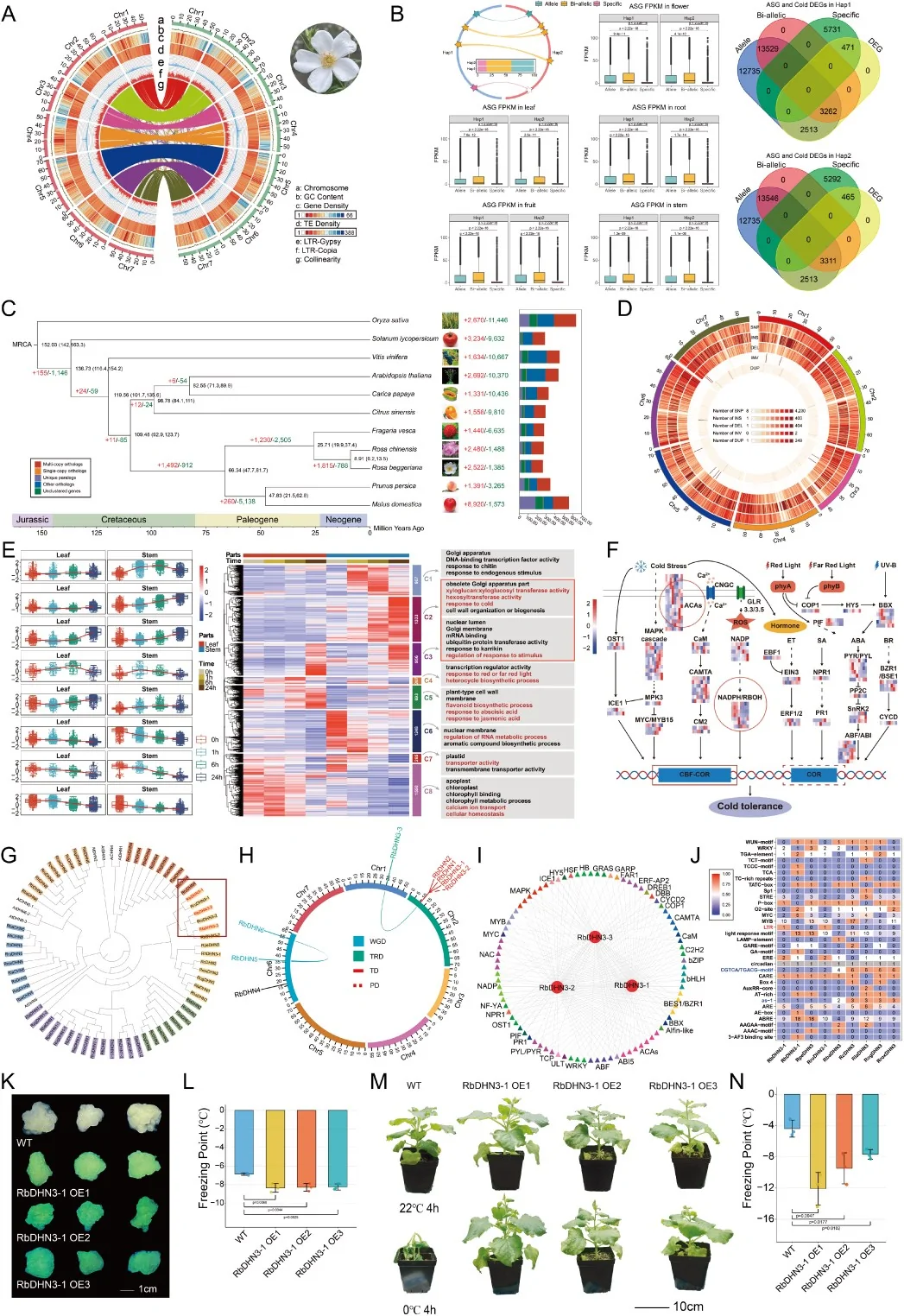
The *R. beggeriana* genome. (A) Genomic landscape of *R. beggeriana*. (a) Chromosomes, green Hap1, red Hap2, centromere gray and telomere black module, (b) Guanine-Cytosine content, (c) gene density, (d) TE density, (e) Gypsy LTR density, (f) Copia LTR density, and (g) synteny regions. (B) Legend of alleles, Bi-allelic and haplotype-specific genes, the expression patterns of various tissues, and the DEGs in response to low-temperature of two haplotypes. (C) Phylogenetic tree and divergence times of *R. beggeriana* and another 10 species. (D) Circos plot of SVs information between the *R. beggeriana* and *R. chinensis* genomes. (E) Expression analysis of different organs and treatment in response to low-temperature. (F) Schematic representation of the backbone pathways of low-temperature response and the expression patterns of key genes. (G) Phylogenetic tree of the DHN gene family in rose (Cold-resistant rose species including *R. beggeriana*, *R. rugosa*, *R. persica*, and *R. hugonis*. Cold-sensitive rose species including *R. chinensis*, *R. roxburghii*, *R. laevigata*, *R. gigantea*, and *R. banksiae*.), with *A. thaliana* as outgroup. (H) The duplication categories of DHN genes in *R. beggeriana*. (I) Network analysis between the DHN genes, transcription factors, and low-temperature response genes in the leaf and stem of *R. beggeriana*. (J) The cis analysis of the promoter of DHN3 in roses. (K) The GFP protein fluorescence of *RbDHN3-1* overexpressing transgenic rose suspension cells. (L) The freezing point of *RbDHN3-1* overexpressing rose suspension cells and Wild Type (WT). (M) Phenotype of *RbDHN3-1* overexpressing tobacco lines and WT under after low-temperature stress for 4 h. (N) The freezing point of *RbDHN3-1* overexpressing tobacco lines and WT.

In addition to sequencing the genome of *R. beggeriana*, we also collected genomes from 10 plant to explore the evolution of *R. beggeriana* (Fig. 1C). Initially, we inferred the polyploidization events experienced by the studied species. *R. beggeriana* underwent whole-genome triplication (γ) and duplication (WGD) events, with an ancient WGD event shared with *R. chinensis* prior to species divergence ~8.91 million years ago. To address the adaptation of species-specific ecological characteristics in *R. beggeriana*, we identified 1,917 unique gene families in *R. beggeriana*, which were significantly enriched in biological processes including ‘root development’ and ‘response to stress’. Additionally, 206 gene families experienced expansion events, with functions primarily focused on ‘response to stress’ and ‘heterocyclic compound binding’, processes crucial for phenotypic adaptation during speciation. Numerous structural variations (SVs) were identified by comparative genomics analysis between *R. beggeriana* and *R. chinensis*, with genes gained via these SVs in *R. beggeriana* being enriched for functions related to ‘response to chitin’ and ‘response to stress’ (Fig. 1D). Stress-responsive functions of SV-gained genes were analyzed by integrating transcriptomic data, revealing that ~30% of these genes (e.g. *ZAT12*, *NCED1*, *MAPK7*, *14-3-3-like*, *MYB17*, etc.) were involved in low-temperature responses associated with transcription, ‌Abscisic Acid‌ (ABA) biosynthesis, kinase signaling, and stress protection. In summary, the evolution of root elongation, stress tolerance, and insect defense in *R. beggeriana* has enhanced its habitat adaptation and shaped its species-specific traits.

To dissect the low-temperature response mechanisms of *R. beggeriana*, we performed time-course transcriptomic analysis of leaf and stem tissues under 4°C treatment. A total of 7160 differentially expressed genes (DEGs) were identified by comparing cold-treated samples with controls across different time points and tissues (Fig. 1E). Weighted gene co-expression network analysis and time-ordered gene co-expression network were employed to unravel key regulatory networks involved in low-temperature response, encompassing Ca^2+^ signaling, reactive oxygen species homeostasis, and phytohormone crosstalk [4] (Fig. 1F). An evolutionary expansion of the *ACAs* and *NADPH* genes contributes to their crucial function in the adaptation of *R. beggeriana* to low temperature. Subsequently, we confirmed that cold-regulated genes (*COR*) in *R. beggeriana* are regulated via both the canonical ICE-CBF-COR pathway and CBF-independent pathways, which constitute the core regulatory framework of low-temperature tolerance [5]. We prioritized dehydrins (DHNs), core members of *COR*, which confer freezing tolerance to plants by stabilizing intracellular macromolecules and modulating membrane fluidity. To address the function of DHN3 in *R. beggeriana*, we revealed distinct duplication categories in species-specific evolution and alterations in gene expression regulation of *RbDHN3* within nine sequenced rose species (Fig. 1G and H). Low-temperature-responsive (LTR) motifs were present only in the promoters of DHN3 in cold-resistant *R. beggeriana* and *R. rugosa* among all species (Fig. 1I and J). The core functional regions of DHN3 are highly conserved, whereas differences in cis-element composition may underlie the distinct cold tolerance patterns observed among roses. We conducted the transgenic materials via overexpressing the *RbDHN3-1* gene in *R. hybrida* ‘Samantha’ suspension cells and tobacco. Phenotypic and physiological assessments demonstrated that *RbDHN3-1* overexpression lines exhibited markedly enhanced cold tolerance by lowering the freezing point and increasing the activities of key antioxidant enzymes of material compared to the wild-type (Fig. 1K–N).

In conclusion, we report the first high-quality, haplotype-resolved genome assembly of *Rosa beggeriana*, which comprehensively elucidates its evolutionary adaptations to habitat conditions and the molecular mechanisms underlying cold tolerance. The assembly and findings presented herein facilitate further investigations into cold tolerance-associated physiological and biochemical traits in roses, and will advance the molecular breeding of cold-tolerant modern roses.

## Data Availability

The data that support the findings of this study have been deposited into CNSA with accession number CNP0008663.

## References

[1] Raymond O, Gouzy J, Just J. et al. The *Rosa* genome provides new insights into the domestication of modern roses. Nat Genet. 2018;50:772–7 10.1038/s41588-018-0110-3 PMC5984618 29713014

[2] Chen F, Su L, Hu S. et al. A chromosome-level genome assembly of rugged rose (*Rosa rugosa*) provides insights into its evolution, ecology, and floral characteristics. Hortic Res. 2021;8:141 10.1038/s41438-021-00594-z PMC8213826 34145222

[3] Cheng B, Zhao K, Zhou M. et al. Phenotypic and genomic signatures across wild *Rosa* species open new horizons for modern rose breeding. Nat Plants. 2025;11:775–89 10.1038/s41477-025-01955-5 40186008

[4] Feng S, Liu Z, Chen H. et al. PHGD: An integrative and user-friendly database for plant hormone-related genes. iMeta. 2024;3:e164 10.1002/imt2.164 PMC10989150 38868516

[5] Zhang H, Zhu J, Gong Z. et al. Abiotic stress responses in plants. Nat Rev Genet. 2022;23:104–19 10.1038/s41576-021-00413-0 34561623

